# Inflence of clinical and genetic characteristics on ability to achive and maintain remission in JIA patients on etanercept treatment

**DOI:** 10.1186/1546-0096-12-S1-P48

**Published:** 2014-09-17

**Authors:** Dragana S Lazarevic, Jelena Vojinovic, Gordana Susic, Jelena Basic

**Affiliations:** 1 Pediatric Rheumatology, Clinic of Pediatrics, Clinical Center Nis, Nis, Serbia; 2 Pediatric Rheumatology, Institute of Rheumatology , Belgrade, Serbia; 3 Institute of Biochemistry, Faculty of medicine, Nis, Serbia

## Introduction

There are no official published recommendations how and when to stop treatment with biologics when remission achieved.

## Objectives

Primary objective of this study was to evaluate influence of JIA subtype and duration of metotrexate (MTX) and steroids treatment on time to achieve and ability to maintain remission in JIA patients on biologic treatment. The second aim was to establish if there is contribution of tumor necrosis factor ά (TNFά–308) promoter and FokI vitamin D receptor (VDR) polymorphism on clinical outcome and possibility to discontinue treatment in JIA patients treated with biologics.

## Methods

68 JIA patients treated with etanercept from Serbian biologic registry were included and retrospective data analysis performed. Genomic DNA was extracted from blood samples and TNFά–308 promoter and FokI VDR polymorphism was evaluated using the PCR-RFLP method. Disease subtypes, activity and treatment efficacy were collected during six years follow-up period in intervals after commencing etanercept: 6 months, 1 year and annually thereafter. Disease remission, as a condition to stop biologic treatment, was defined using Wallace and all criteria [1].

## Results

At enrolment JIA patients mean age were 183.34±60,58 months, disease duration 70.29±44,57 months, average dose of MTX 13.85±4.47 mg/m2/week. Etanercept treatment could be stopped after 42.66±21.64 months with sustained remission during the next 30.33±21.04 months. Therapy resistant patients required higher doses of MTX for a longer period, with statistically significance in systemic JIA (15.97±3.56 vs. 13.15±4.55, p=0.016). Remission in this patients was shorter and they needed retreatment with biologics (16.31±18.55 vs. 35.80±19.53, p=0.001) due to disease worsening. Treatment inefficacy was present in systemic JIA with the longest etanercept treatment 59.50±5.97 and shortest remission 17.67%±9.82, while etanercept therapy was the most effective in RF- JIA patients. There was no statistically significant difference in cumulative dose of steroids in different JIA subtypes. The distribution of TNFά–308 (GG, GA, AA) and FokI VDR genotypes (FF,Ff, ff) was not significantly different among JIA subtypes. After six years follow up period 37(54.5%) patients were in remission (20 patients with FF+GA and 17 patients with Ff+GA polymorphism). Associate presence of FF+GA genotype was present more frequently in patients who needed longer treatment and have had shorter remission time.

## Conclusion

JIA patients needing higher MTX doses to control disease and have associated presence of GA+FF polymorphisms (for TNFά–308 promoter and FokI VDR, respectively) have less chances to achieve and sustain remission off biologics, especially in systemic JIA..

## Disclosure of interest

None declared.
